# Longitudinal assessment and predictors of subjective taste change after hematopoietic cell transplantation (HCT)

**DOI:** 10.1007/s00520-026-10831-7

**Published:** 2026-06-03

**Authors:** Allan J. Hovan, Michael T. Brennan, Bengt Hasséus, Judith E. Raber-Durlacher, Marie-Charlotte Huysmans, Alexa M. G. A. Laheij, Stephanie J. M. van Leeuwen, Karin Garming Legert, Scott Isom, David M. Kline, Nicole M. A. Blijlevens, Jan-Erik Johansson, Inger von Bültzingslöwen

**Affiliations:** 1 Department of Oral Oncology and Dentistry, British Columbia Cancer, Vancouver, BC Canada; 2 Department of Oral Medicine/Oral & Maxillofacial Surgery, Wake Forest University School of Medicine, Atrium Health Carolinas Medical Center, Charlotte, NC USA; 3 Department of Oral Medicine and Pathology, Institute of Odontology, The Sahlgrenska Academy, University of Gothenburg, Gothenburg, Sweden; 4 Department of Oral Medicine, Academic Centre for Dentistry Amsterdam (ACTA), University of Amsterdam and VU University, Amsterdam, The Netherlands; 5 Department of Oral and Maxillofacial Surgery, Amsterdam UMC, Vrije Universiteit Amsterdam, Amsterdam, The Netherlands; 6 Department of Dentistry, Radboud university medical center, Nijmegen, The Netherlands; 7 Department of Dental Medicine, Karolinska Institutet, Huddlinge, Sweden; 8 Division of Public Health Sciences, Department of Biostatistics and Data Science, Wake Forest University School of Medicine, Winston-Salem, NC USA; 9 Department of Hematology, Radboud university medical center, Radboud Institute for Health Sciences, Nijmegen, The Netherlands; 10 Section of Hematology, the Sahlgrenska Academy, University of Gothenburg, Gothenburg, Sweden; 11 Department of Oral Microbiology and Immunology, Institute of Odontology, The Sahlgrenska Academy, University of Gothenburg, Gothenburg, Sweden

**Keywords:** Dysgeusia, Taste changes, Hematopoietic cell transplant, HCT, Incidence, Risk factors

## Abstract

**Purpose:**

The aims of this investigation within the Orastem study were to describe the prevalence of subjective taste change, levels of patient-reported distress associated with taste change, and factors predicting taste change after hematopoietic cell transplantation (HCT) with a particular focus on factors predictive of severe and/or persistent (present at 12 months) taste change.

**Methods:**

Altogether, 238 patients at five cancer centers were followed during HCT hospitalization and up to 12 months post-HCT. Patients with taste change were compared to non-affected patients with respect to gender, age, transplant type, previous chemotherapy, conditioning regimen(s), oral mucositis, pre-HCT salivary flow, oral hygiene status and, in allogeneic HCT recipients, chronic oral graft-versus-host disease (GVHD). Distress from taste change was also evaluated.

**Results:**

During hospitalization, taste change was reported by over one-third (81/238) of patients to an open question on oral symptoms; of these, 41% reported taste change as their worst/most bothersome oral symptom. To a specific question on distress due to taste change, 76% of all patients reported some level of distress. Taste change from previous chemotherapy and full intensity conditioning were risk factors for severe taste change during hospitalization. At three months, allogeneic transplant patients reported a higher rate of severe taste change compared to autologous transplant patients. Increased age predicted severe taste change at 6 and 12-month follow-up. At twelve months, 13.1% of patients reported distress from taste change.

**Conclusion:**

This study confirmed taste change as a common clinical complaint post-HCT. Taste change remained a significant issue for a small subset of patients at 12 months. Further research is required to develop effective preventive and management strategies for this common oral side-effect post-HCT.

**Supplementary Information:**

The online version contains supplementary material available at 10.1007/s00520-026-10831-7.

## Introduction

Taste change is a commonly reported side-effect of cancer therapy and haematopoietic cell transplantation (HCT) [1–4]. Taste change is also reported in non-cancer patients with potential causes including vitamin and mineral deficiencies, oral infections and other less well-understood central mechanisms [5]. Regardless of aetiology or medical status, the impact of taste change can be significant in terms of the patient’s nutritional status and overall quality of life [6, 7].

Conditioning chemotherapy ± total body irradiation (TBI) for HCT with or without graft-versus-host disease (GVHD) prophylaxis is a well-established and potentially curative treatment for many hematological malignancies as well as other malignant and non-malignant disorders [8, 9]. There is a lack of knowledge regarding the prevalence, severity and factors predictive of oral complications. To our knowledge, taste change has not been widely studied in large, multicentre prospective clinical trials in the HCT population with a wide range of medical diagnoses and conditioning regimens.

Patients undergoing HCT represent a distinct population in which taste is potentially impaired by numerous factors including conditioning regimen-related mucosal injury, prophylactic and supportive medications and changes in the oropharyngeal and gastrointestinal microbiome [10, 11].

The contributors to taste change may differ between autologous and allogeneic transplant recipients. Early taste disturbances after autologous transplant are often caused by direct conditioning regimen-related oral mucosal injury from chemotherapy with or without TBI. Late effects on taste following autologous transplant appear to be largely a function of residual conditioning regimens and unwanted side-effects of supportive medications [12]. High-dose melphalan and other melphalan-containing chemotherapy regimens (e.g. BEAM) are widely used as conditioning chemotherapy, particularly in the autologous setting. Previous studies have shown that both high-dose melphalan and BEAM-like regimens are independent risk factors for taste change [13, 14] which may be related to the relatively high levels of melphalan secreted in whole saliva [15].

For patients receiving an allogeneic transplant, taste change appears to be a more complex and persistent problem. This is likely due to the increased frequency of infections [16], the routine use of immunosuppressant medications for both prevention and management of GVHD and other alloreactive complications unique to this type of transplant [17, 18].

The purpose of this study was to describe the prevalence of subjective taste change, levels of patient-reported distress associated with taste change, and factors which may predict risk of taste change in HCT patients with a particular focus on factors which predict severe and/or persistent (present at 12 months) taste change. These results are part of a large international prospective multicenter observational study (Orastem) involving patients undergoing either autologous or allogeneic HCT for a variety of haematologic malignancies.

## Methods

Patients were recruited at Sahlgrenska University Hospital Gothenburg and Karolinska University Hospital Huddinge, Stockholm, Sweden; Atrium Health Carolinas Medical Center, Charlotte, NC, USA; BC Cancer, Vancouver, BC, Canada; Amsterdam UMC, University of Amsterdam, Amsterdam, the Netherlands and Radboud university medical center, Nijmegen, the Netherlands. The data from the two Swedish centers were merged and considered as one site.

Patients ≥ 18 years old scheduled for a conditioning regimen followed by either autologous or allogeneic HCT were included in the study. The Orastem study protocol has been previously described and involved five study phases starting with a baseline assessment before HCT and concluding with a 12-month post-HCT assessment [19]. All examiners were calibrated at the start of the study. Ethics approval was obtained from all participating institutions and informed consent was obtained from all participants in the study.

A pre-treatment assessment reviewed medical conditions, planned chemo- and radiation therapy regimens, objective documentation of oral conditions, measurement of whole salivary flow rate and a review of current lab values. Patients were interviewed at baseline regarding any subjective oral complaints they were experiencing. Information on oral health-related habits such as history of professional dental care as well as frequency of both tooth brushing and dental flossing was collected. In terms of oral hygiene status, patients were divided into groups with < 20% or ≥ 20% of teeth with visible plaque as poor oral hygiene was viewed as a potential independent risk factor for taste change [20, 21].

A variety of conditioning regimens were used in HCT: non-myeloablative, reduced intensity and full intensity. In the analysis, a comparison was made between patients who received non-myeloablative/reduced intensity conditioning and patients who received full intensity conditioning in terms of taste change.

Oral mucositis was measured using the World Health Organization (WHO) toxicity scale (score 0–4) [22] and was recorded pre-HCT, during hospitalization and at each follow-up clinical appointment. Patients were divided into two groups based on a WHO mucositis score of < 2 vs ≥ 2. The worst mucositis score during hospitalization was used for statistical analysis.

Stimulated whole saliva was measured pre-HCT by having patients chew on paraffin wax for 5 min [23]. Salivary flow rate was recorded as mL/min [24]. For the purposes of analysis, patients were divided into two groups (< 1 mL/min and ≥ 1 mL/min), based on mean flow rates of stimulated saliva [25].

Starting 1–3 days after hematopoietic cell infusion, a bedside assessment was scheduled 3 days per week until the absolute neutrophil count (ANC) reached > 0.5 × 10^9^/L. Patients were regularly asked if they were experiencing any oral symptoms. Patients were then asked to specifically describe any oral symptoms and to rate the worst/most bothersome oral symptom using a numeric rating scale from 0–10. For patients reporting taste change as their worst/most bothersome symptom, a taste change severity score was calculated using the highest score (0–10) during hospitalization. Patients were also asked about any impact taste change was having on their ability to eat solid foods and/or swallow liquid/pureed foods.

A patient questionnaire was completed 3 days per week in which patients were asked more specifically to rate any taste change and reduction in taste sensitivity during the past 24 hours on a 4-point scale (“not at all”, “a little”, “quite a bit” or “very much”). Similarly, patients were asked to rate any distress caused by taste change over the previous 24 hours using a 5-point scale (“not at all”, “a little”, “some”, “a lot” or “severely”). The percentage of patients reporting severe (“a lot”/”severely”) distress during the last 24 hours was calculated during hospitalization and at all subsequent follow-up appointments.

Once ANC had reached > 0.5 × 10^9^/L, patients with any ongoing oral mucosal or dental issues were followed in either an inpatient or outpatient setting. Additional visits for urgent care for acute oral issues after hospitalization (Phase IV) were documented.

Autologous transplant patients were followed up in person and by patient questionnaire at 3 months and by patient questionnaire one-year post-HCT to identify any long-term side-effects. Allogeneic transplant patients were followed up in person and by patient questionnaire at 3, 6 and 12 months post-HCT. The patient questionnaire used during hospitalization was applied at all follow-up appointments.

The 3-, 6-and 12-month examinations of allogeneic transplant patients also included an evaluation of clinical signs of chronic oral GVHD as this may predispose the patient to subjective taste change [26, 27]. Chronic oral GVHD is characterized clinically by the presence of lichenoid mucositis, salivary dysfunction and tissue sclerosis. Symptoms may include oral pain, mucosal sensitivity to previously tolerated substances, dry mouth and limited oral opening [28, 29].

**Fig. 1:**
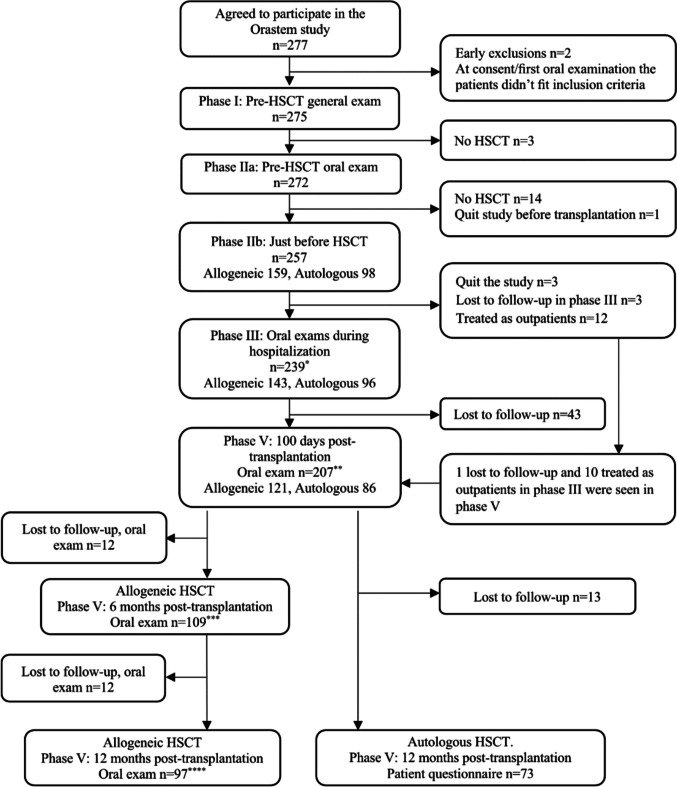
Orastem flowchart, showing number of patients in each phase of the study [24]. *195 patients answered questionnaire, **161 patients answered questionnaire, ***92 patients answered questionnaire, ****96 patients answered questionnaire. HSCT = HCT: hematopoietic cell transplantation

The various phases of the study are shown in Fig. 1 as first published by Skallsjö et al. [24].

### Statistical analyses

Descriptive statistics were used to show patient characteristics prior to HCT and at follow-up periods. Comparisons for those whose information was available at follow-up were compared to those without follow-up information using exact tests, t-tests, and Kruskal Wallis tests depending on the type and distribution of the variables to explore potential differential loss to follow-up. The primary aim was to investigate associations between patient characteristics and severe taste change during hospitalization and subsequent follow-up assessments. To summarize the incidence of severe taste change during hospitalization, a patient’s most severe taste change in 24 hours during their stay was used to represent their taste change during hospitalization (0–35 days post HCT). Taste change during hospitalization, 3, 6 and 12 months was then categorized as either “not at all”/”a little” or “quite a bit”/”very much” with the latter group being considered to have severe taste change symptoms.

Hospitalization information was treated separately from the long-term follow-up data since the questions used to collect the information were slightly different. A series of longitudinal generalized estimating equations (GEE) models with a logit link and binomial variance function was used to identify characteristics associated with severe taste change and to account for some pre-treatment patient characteristics (gender, age, treatment type, clinical site) as well as days from HCT. Odds ratios and associated p-values from these models with empirical standard errors were used to determine associations of characteristics with severe taste change. These analyses are considered exploratory and therefore no adjustments for multiple comparisons were used. All analyses were done using SAS version 9.4 (SAS Institute, Cary, NC).

## Results

### Before hospitalization

Baseline demographics of patients prior to hospitalization are shown in Table 1. Of the 257 patients who participated in the Phase IIb pre-HCT evaluation, 246 reported a history of previous chemotherapy. Of the 246 patients, over half (*n*=148; 60.2%) reported oral complications from previous chemotherapy. Overall, 75 of all Phase IIb patients (29.3%) specifically reported taste change from previous chemotherapy to an open question on oral complications from chemotherapy, making it the most commonly reported pre-HCT oral complication followed by dry mouth (26.2%) and oral mucositis (12.1%).

**Table 1:** Baseline demographics prior to hospitalization

	*N* (%)
Number of patients	257
Location	
Gothenburg/Stockholm	32 (12.4%)
Vancouver	53 (20.6%)
Amsterdam (AMC)	42 (16.3%)
Nijmegen (RUNMC)	77 (30.0%)
Charlotte	53 (20.6%)
Age, mean years (S.D.)	53.5 (12.5)
Age, median years (25th perc; 75th perc)	56.0 (48.0; 63.0)
Gender	
Male	147 (57.2%)
Female	110 (42.8%)
Medical diagnosis requiring transplant	
AML (Acute myelogenous leukemia)	62 (24.1%)
ALL (Acute lymphoblastic leukemia)	17 (6.6%)
Lymphoma	41 (16.0%)
CLL (Chronic lymphocytic leukemia)	8 (3.1%)
MDS (Myelodysplastic syndrome)	17 (6.6%)
CML (Chronic myelogenous leukemia)	8 (3.1%)
Myelofibrosis	13 (5.1%)
Myeloproliferative disorder	1 (0.4%)
SAA (Severe aplastic anemia)	4 (1.6%)
Multiple Myeloma	75 (29.2%)
Other	11 (4.3%)
Regimen Intensity	
Full intensity (FIC)/myeloablative	134 (52.1%)
Reduced intensity (RIC)	92 (35.8%)
Non-myeloablative (NMA)	31 (12.1%)
Type of Transplant	
Allogeneic	159 (61.9%)
Autologous	98 (38.1%)
Conditioning regimen	
BEAM (carmustine, cytarabine, etoposide, melphalan)	19 (7.4%)
Busulfan/cyclophosphamide	13 (5.1%)
Busulfan/fludarabine	30 (11.7%)
Cyclophosphamide	6 (2.3%)
Cyclophosphamide/total body irradiation	23 (8.9%)
Fludarabine/treosulfan	4 (1.5%)
Fludarabine/total body irradiation	33 (12.8%)
Fludarabine/cyclophosphamide/total body irradiation	43 (16.7%)
Melphalan 140	4 (1.6%)
Melphalan 200	73 (28.4%)
Others (≤ 2 each)	9 (3.5%)
Use of Melphalan in conditioning regimen	
No	159 (61.9%)
Yes	98 (38.1%)

### During hospitalization

Of the 257 patients who had undergone a pre-HCT evaluation just before HCT (Phase IIb), 239 were initially included in the clinical evaluation during hospitalization. Exclusions included 12 patients who were treated as outpatients, 3 patients who quit the study and 3 patients lost to follow-up (see Fig. 1). One more patient was excluded as data collection was not possible due to intensive care and subsequent palliative care. Hence, 238 patients were reported in the clinical analysis (Table 2). At one of the participating centres, patient questionnaires were not completed for a subset of their patients during hospitalization, 3-month and 6-month evaluations, leaving 195 patients answering the patient questionnaire during hospitalization. One patient was too sick to complete the questionnaire for the first two weeks. Thus, 194 patients were followed-up by patient questionnaire during hospitalization. In addition, at all follow-up evaluations, there were a few individual patients (Fig. 1) per centre who were either lost to follow-up, did not complete a questionnaire or who left certain questions within the questionnaire unanswered. These factors led to minor discrepancies in total patients reported for any particular follow-up period.

**Table 2:** Characteristics of patients reporting taste change during hospitalization (Phase III)

	No Taste Change *N* (%)	Taste Change *N* (%)	*p*-value
Number of patients	157	81	
Type of Transplant			**0.0156**
Allogeneic	85 (54.1%)	57 (70.4%)	
Autologous	72 (45.9%)	24 (29.6%)	
Regimen Intensity			0.3882
Full intensity/myeloablative	88 (56.1%)	43 (53.1%)	
Reduced intensity	55 (35.0%)	34 (42.0%)	
Non-myeloablative	14 (8.9%)	4 (4.9%)	
Worst WHO Mucositis score during hospitalization			**0.0076**
< Grade 2	109 (69.4%)	42 (51.9%)	
≥ Grade 2	48 (30.6%)	39 (48.1%)	
Dental plaque score			0.9642
< 20% of teeth	99 (70.7%)	49 (71.0%)	
≥ 20% of teeth	41 (29.3%)	20 (29.0%)	
Stimulated salivary flow at baseline			0.8815
< 1 mL/min	64 (42.4%)	33 (43.4%)	
≥ 1 mL/min	87 (57.6%)	43 (56.6%)	
Toothbrushing habits at baseline			0.2470
< twice per day	32 (21.1%)	22 (27.8%)	
≥ twice per day	120 (78.9%)	57 (72.2%)	
Flossing habits at baseline			0.6073
< once per day	72 (48.3%)	41 (51.9%)	
≥ once per day	77 (51.7%)	38 (48.1%)	
Dental visits			0.1048
Never or only for acute problems	33 (21.3%)	25 (30.9%)	
Routinely	122 (78.7%)	56 (69.1%)	
Prior chemotherapy			0.4407
No	7 (4.5%)	2 (2.5%)	
Yes	149 (95.5%)	79 (97.5%)	
Taste change from prior chemotherapy			** < 0.0001**
No	122 (78.2%)	42 (51.9%)	
Yes	34 (21.8%)	39 (48.1%)	

Overall, 81/238 (34%) reported taste change as an oral complication at some point during their hospitalization as an answer to an open question on any oral symptoms they were experiencing. Of these 81 patients, approximately half (41/81) reported taste change as their worst/most bothersome oral symptom. This represents 17.2% of all hospitalized patients. The mean taste change severity score was 5.3 (s.d. = 2.9) using the numeric rating scale from 0–10 and calculated using the worst score during hospitalization. Dry mouth (63.9%) and oral pain (24.4%) were more commonly reported as the worst/most bothersome oral symptom during hospitalization. 

To the specific question in the patient questionnaire on taste change in the last 24 hours, 94/194 (48.5%) of patients reported severe taste change (“quite a bit”/”very much”) at some point during hospitalization. Approximately one-third of patients (66/194;34%) reported a severe (“quite a bit”/”very much”) reduction in taste sensitivity in the last 24 hours (Table 3).

**Table 3:** Taste change and taste sensitivity reduction in the last 24 hours reported by patients during the first two weeks (Hospitalization) after hematopoietic cell transplantation and after 3, 6 and 12 Months*

	Hospitalization* *N* (%)		3 months *N* (%)		6 months (allo only) *N* (%)		12 months *N* (%)	
During the past 24 hours, did you have a change in taste?								
Not at all	35 (18.0)		119 (74.4)		70 (78.7)		136 (82.9)	
A little	65 (33.5)	159 (82.0)	29 (18.1)	41 (25.6)	12 (13.5)	19 (21.3)	12 (7.3)	28 (17.1)
Quite a bit	58 (29.9)		10 (6.3)		3 (3.4)		12 (7.3)	
Very Much	36 (18.6)		2 (1.3)		4 (1.5)		4 (2.4)	
During the past 24 h, was your taste sensitivity reduced?								
Not at all	57 (29.4)		132 (82.5)		79 (87.8)		141 (84.9)	
A little	71 (36.6)	137 (70.6)	22 (13.8)	28 (17.5)	4 (4.4)	11 (12.2)	10 (6.0)	25 (15.1)
Quite a bit	46 (23.7)		5 (3.1)		5 (5.6)		10 (6.0)	
Very Much	20 (10.3)		1 (0.6)		2 (2.2)		5 (3.0)	

^*^Using worst response during hospitalization

**Fig. 2:**
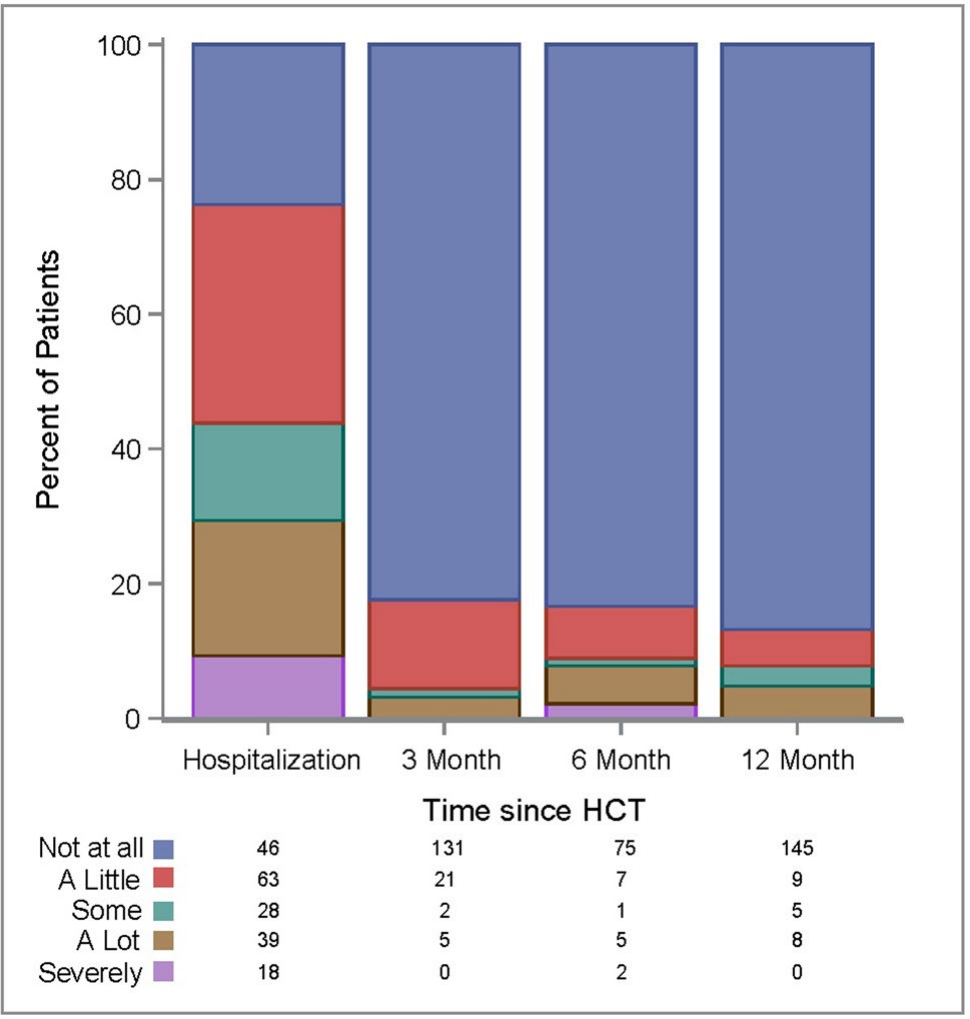
Distress due to taste change after hematopoietic cell transplantation

Furthermore, 148/194 (76.3%) of patients reported any level of distress due to taste change during hospitalization and, of these, 57/194 (29.4%) rated their distress as severe (“a lot”/”severely”) (Fig. 2).

In total, 137/238 (57.6%) of patients reported an inability to eat solid foods and/or swallow liquid/pureed foods at some point during their hospitalization. 28/238 (11.8%) patients reported that they would be able to eat solid foods if not for the taste change symptoms they were experiencing; 12/238 (5%) patients reported that they would be able to swallow liquid/pureed food if not for their taste change symptoms.

**Fig. 3:**
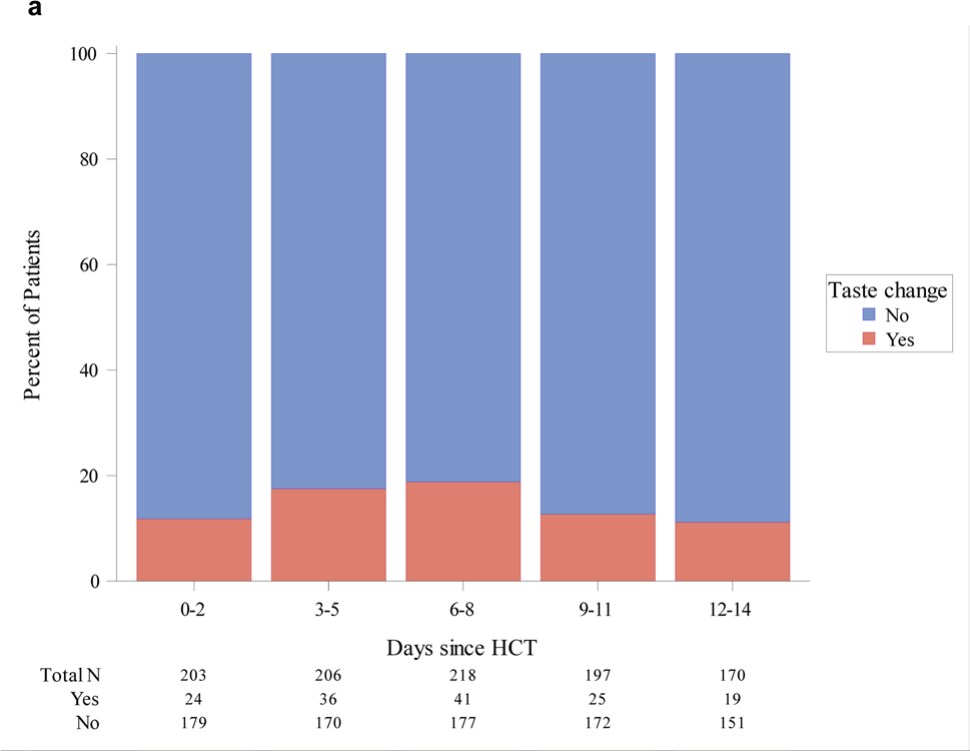
Frequency of hospitalized patients reporting taste change as an answer to an open question on oral symptoms*, * Calculations were done in 3-day intervals after hematopoietic cell transplantation. Each patient was counted only once during each 3-day interval using the highest value over the 3-day interval

**Fig. 4:**
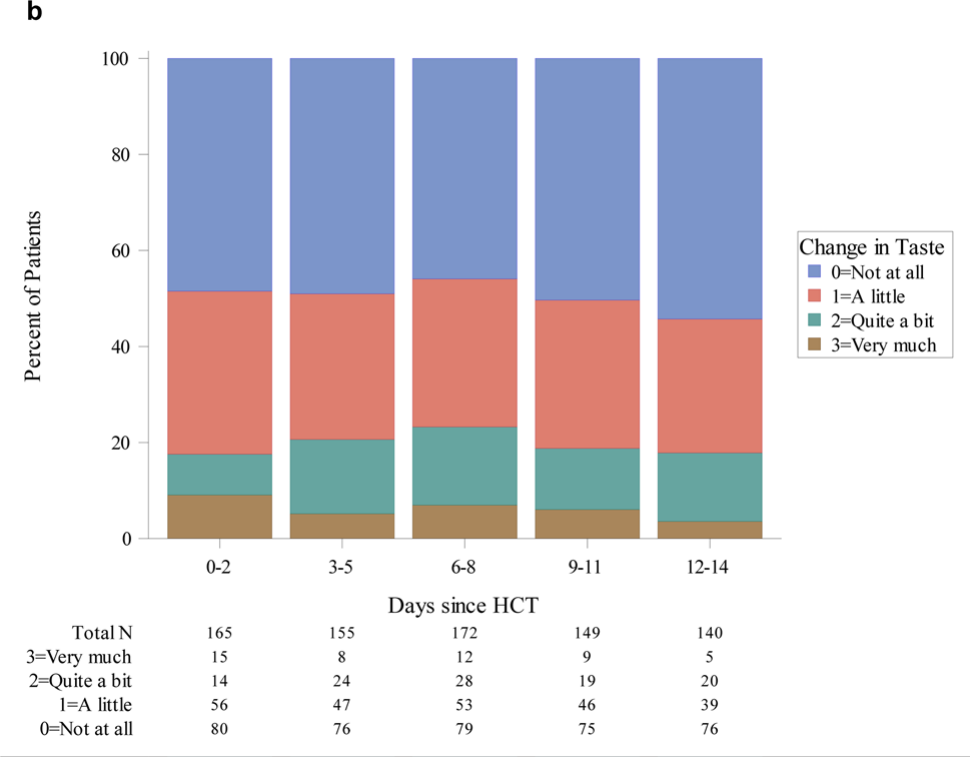
Frequency of hospitalized patients reporting taste change as an answer to a specific question on taste change during the last 24 hours*, *Calculations were done in 3-day intervals after hematopoietic cell transplantation. Each patient was counted only once during each 3-day interval, using the highest value over the 3-day interval

Evaluating subjective taste change over time during hospitalization, symptoms were most commonly reported in the two weeks immediately following HCT when most patients were profoundly neutropenic (Figs. 3 and 4). Although somewhat variable, the period of neutropenia typically resolved within the first 2 weeks post-HCT and taste change symptoms tended to diminish in the majority of patients after 2 weeks.

Taste change during hospitalization was associated with allogeneic transplant patients (*p* = 0.0156), patients who experienced WHO ≥ Grade 2 oral mucositis (*p* = 0.0076) and patients who experienced taste change from previous chemotherapy (*p* < 0.0001) (Table 2). Salivary flow < 1 mL/min pre-HCT, visible plaque levels on > 20% of teeth, frequency of pre-HCT dental visits, gender and increased age did not predict risk of any level of taste change during hospitalization. There was no GVHD prophylaxis regimen shown to put patients at higher risk for taste change post-HCT.

Using univariate analysis, melphalan conditioning initially appeared to predict risk of severe taste change during hospitalization (Supplement 1). However, after multivariate analysis, the only factors shown to predict risk of severe taste change during hospitalization were patients who had experienced taste change from previous chemotherapy (*p* = 0.0224) and patients who had received full intensity conditioning (*p* = 0.0544).

### 3-month follow-up

At the 3-month follow-up, a total of 207 patients (121 allogeneic; 86 autologous) completed oral exams and a total of 161 patients completed all or most of the questions in the questionnaire.

Patients were asked about any taste change or reductions in taste sensitivity in the last 24 hours. Overall, 41/160 (25.6%) of patients reported some level of taste change and 28/161 (17.4%) reported some reduction in taste sensitivity. 12/160 (7.5%) of patients reported their taste change as severe; 6/161 (3.7%) of patients reported their reduction in taste sensitivity as severe (Table 3). 28/159 (14.4%) of patients reported some level of distress from taste change and, of these, 5 patients rated their distress as severe (Fig. 2).

Chronic oral GVHD was noted in 14/119 (11.8%) of allogeneic transplant patients. Among these 14 patients, 5 also reported severe taste change symptoms. Overall, of the patients with severe taste change at 3 months, the rate was higher in the oral GVHD group (35.7%) than in the non-oral GVHD group (21.9%) (Supplement 2).

After multivariate adjustment, the only factors shown to increase risk for severe taste change or severe reduction in taste sensitivity at 3-month follow-up were increased age (*p* = 0.0015) and allogeneic transplant (*p* = 0.0365). The other factors studied did not predict increased risk of severe taste change (Supplement 3).

### 6-month follow-up

At the 6-month follow-up (allogeneic transplant patients only), a total of 109 patients completed an oral exam and 92 patients completed all or most of the questions in the questionnaire.

Patients were again asked about any taste change or reductions in taste sensitivity. 19/89 (21.3%) reported some level of taste change in the last 24 hours and 11/90 (12.2%) reported some reduction in taste sensitivity during the past 24 hours. 7/89 (7.9%) of patients reported their taste change as severe and 7/90 (7.8%) rated their reduction in taste sensitivity as severe (Table 3). 15/90 (16.7%) of patients reported some level of distress from taste change and, of these, 7 patients rated their distress as severe (Fig. 2).

Of the 108 allogeneic transplant patients examined at 6-month post-HCT follow-up, chronic oral GVHD was noted in 29/108 (26.8%) of the patients. Severe taste change was reported in 8/29 of these patients. The rate of severe taste change was higher in the GVHD group (27.6%) than in the non-GVHD group (12.7%) (Supplement 2).

After multivariate adjustment, increased age was the only factor shown to predict risk for severe taste change at 6-month follow-up (*p* = 0.0070) (Supplement 3).

### 12-month follow-up

Nine patients required additional visits to address acute oral-care issues between hospitalization and 12-month follow-up. None of these visits were related specifically to taste change symptoms.

At the 12-month follow-up, 97 allogeneic patients completed oral exams and 169 patients (96 allogeneic and 73 autologous) completed all or most of the questions in the questionnaire. 28/164 (17.1%) of patients reported some level of taste change in the last 24 hours and 25/166 (15.1%) reported a reduction in taste sensitivity in the last 24 hours. 16/164 (9.7%) rated their taste change as severe and 15/166 (9.0%) rated their reduction in taste sensitivity as severe (Table 3). 22/167 (13.1%) of patients reported some level of distress from taste change and, of these, 8 patients reported their distress as severe (Fig. 2).

Chronic oral GVHD was noted in 23/96 (23.9%) of allogeneic transplant patients and the prevalence of severe taste change was higher in the oral GVHD group (17.4%) than in the non-oral GVHD group (11.0%) (Supplement 2).

After multivariate adjustment, the only factor that persisted as a predictor of risk for severe taste change at 12-month follow-up was increased age (*p* = 0.0145) (Supplement 3).

## Discussion

To our knowledge, the Orastem study is the largest prospective multicentre observational study specifically examining the various oral side-effects of HCT in an international cohort of autologous and allogeneic transplant patients followed from pre-HCT hospital admission until 12 months post-HCT.

The specific goals of this investigation within the Orastem study were to describe the prevalence of subjective taste change, the levels of patient-reported distress associated with taste change, and factors predicting risk of developing severe and/or persistent taste change as part of HCT.

In terms of the prevalence of taste change symptoms, a significant percentage of patients (75/246; 29.3%) entered the study reporting taste change from previous chemotherapy. Increases in patient-reported taste change and reduction in taste sensitivity were noted during hospitalization. At 12-month follow-up, 19/114 (16.6%) of patients continued to report taste change and 16/114 (14%) reported reductions in taste sensitivity. Taste change was rated as severe in 13/19 patients and reduction in taste sensitivity was rated as severe in 11/16 patients.

Taste change appeared to cause significant levels of distress in some patients, most particularly during hospitalization. During hospitalization, 148/194 (76%) of patients reported some level of distress from taste change. At 12-month follow-up, 22/167 (13.2%) patients continued to report some level of distress. Of these, 8 patients rated their distress as severe.

Consistent with other published studies [30, 31], our data confirmed that previous chemotherapy increased the risk for overall taste change and taste sensitivity reduction.

Taste change has been reported as a side-effect of high-dose chemotherapy, including melphalan. In our study, melphalan initially appeared to cause an increase in taste change during hospitalization. However, this effect was not seen after multivariate analysis and was not seen during follow-up.

WHO oral mucositis scores > 2 increased risk for severe taste change during hospitalization but this effect was not reported by patients at subsequent follow-up appointments. Allogeneic transplant patients experienced higher rates of taste change during hospitalization and at all follow-up visits. At 3 months, severe taste change was statistically higher in allogeneic transplant patients. Taste change scores were consistently higher in patients with chronic oral GVHD but this did not reach statistical significance at any of the follow-up periods (Supplement Table 2).

Previous studies have indicated that lower salivary levels can be a risk factor for taste change and other symptoms in cancer patients [32, 33]. In our study, no difference was seen between patients with < or ≥ 1 mL saliva/min pre-HCT and taste change.

It is known that differences may exist between objective measures of salivary flow and subjective reporting of xerostomia [34, 35]. In our study, an objective measure of salivary flow at baseline was used to dichotomize patients for analysis. However, all follow-up assessments were based on subjective reporting only. The use of objective measures of salivary flow throughout the course of patient follow-up would have made additional conclusions possible with respect to salivary volume and dysgeusia risk.

Similarly, all measures of taste change were subjective in nature. It was beyond the scope of this study to apply objective measures of dysgeusia (e.g. chemical gustometry) to our patient group. Future studies should consider the use of objective measures of dysgeusia, if possible, to strengthen any conclusions regarding risk factors for dysgeusia in an HCT population.

It is noteworthy that patients who reported taste change symptoms upon entry to the study were not questioned about the severity of their symptoms pre-HCT. Interestingly, none of the 16 patients reporting severe taste change at 12-month follow-up had reported any level of taste change on entry to the study. It therefore appears that de novo cases of severe taste change appeared in a small number of patients, unrelated to pre-HCT symptoms.

The heterogeneity of the patient population studied can be seen as both a strength and weakness of the study. Having patients from a number of countries and institutions increases the applicability of the results. However, regional and/or cultural differences have the potential to bias the results. A much larger study would be required to control for the variability of heterogeneity.

Patients were lost to follow-up (LTFU) over the course of the study and, although statistical adjustments were performed to account for LTFU, it is possible that patients experiencing the highest rates of side-effects (including taste change) are over-represented in the LTFU group. Our data may therefore under-report the true incidence and severity of taste change in the post-HCT study population.

Other limitations of the study include the different follow-up schedules for autologous vs allogeneic transplant patients and the relatively short follow-up period (12 months). It is also noteworthy that no specific questions were asked about changes in smell which have been shown to be linked to taste changes in some patients [36].

In summary, this study confirmed taste change as a common clinical complaint among pre-HCT patients which became more prevalent immediately following HCT. Taste change impacted on the ability to eat and swallow foods during hospitalization and caused significant distress in some patients. While symptoms became less problematic for most patients post-HCT, it remained a significant clinical and psychological issue for a small subset of patients. Older age was the only factor shown to predict severe taste change at 12-month follow-up.

Further research is required to further explore strategies to more effectively prevent and manage this common oral side-effect of HCT therapy.

## Supplementary Information

Below is the link to the electronic supplementary material.

ESM 1 (XLSX 16.7 KB)

ESM 2 (DOCX 14.4 KB)

ESM 3 (DOCX 18.3 KB)

## Data Availability

Research data cannot be shared publicly because all data are stored at and belong to the University of Gothenburg (GU), and there are ethical and legal restrictions on sharing our data set according to Swedish law. Study data contain sensitive patient information that can be connected to individual patients. It may be possible to identify and connect personal information from the data set, even though data are de-identified. All relevant data within the paper are at group level.

## References

[1] Hovan AJ, Williams PM, Stevenson-Moore P, Wahlin YB, Ohrn KEO, Elting LS, Spijkervet FKL, Brennan MT, Dysgeusia Section, Oral Care Study Group, Multinational Association of Supportive Care in Cancer (MASCC)/International Society of Oral Oncology (ISOO) (2010) A systematic review of dysgeusia induced by cancer therapies. Support Care Cancer 18(8):1081–1087. 10.1007/s00520-010-0902-1 20495984 10.1007/s00520-010-0902-1

[2] Dequae C, Raber-Durlacher J, Epstein J, de Vries R, Laheij AMGA (2024) Taste alterations after hematopoietic cell transplantation. Support Care Cancer 32(10):687. 10.1007/s00520-024-08900-w 39320564 10.1007/s00520-024-08900-w PMC11424654

[3] Wickham RS, Rehwaldt M, Kefer C, Shott S, Abbas K, Glynn-Tucker E, Potter C, Blendowski C (1999) Taste changes experienced by patients receiving chemotherapy. Oncol Nurs Forum 4:697–706 10337648

[4] Bressan V, Stevanin S, Bianchi M, Aleo G, Bagnaasco A, Sasso L (2016) The effects of swallowing disorders, dysgeusia, oral mucositis and xerostomia on nutritional status, oral intake and weight loss in head and neck cancers patients: a systematic review. Cancer Treat Rev 45:105–119. 10.1016/j.ctrv.2016.03.006 27010487 10.1016/j.ctrv.2016.03.006

[5] Jafari A, Alaee A, Ghods K (2021) The etiologies and considerations of dysgeusia. A review of literature. J Oral Biosci 63(4):319–326. 10.1016/j.job.2021.08.006 34487857 10.1016/j.job.2021.08.006

[6] Epstein JB, Smutzer G, Doty RL (2016) Understanding the impact of taste changes in oncology care. Support Care Cancer 24(4):1917–1931. 10.1007/s00520-016-3083-8 26820877 10.1007/s00520-016-3083-8

[7] Gunn L, Gilbert J, Nenclares P, Soliman H, Newbold K, Bhide S, Wong KH, Harrington K, Nutting C (2021) Taste dysfunction following radiotherapy to the head and neck: a systematic review. Radiother Oncol 157:130–140. 10.1016/j.radonc.2021.01.021 33545253 10.1016/j.radonc.2021.01.021

[8] Copelan EA (2006) Hematopoietic stem cell transplantation. N Engl J Med 354(17):1813–1826 16641398 10.1056/NEJMra052638

[9] Majhail NS, Farnia SH, Carpenter PA, Champlin RE, Crawford S, Marks DI, Omel JL, Orchard PJ, Palmer J, Saber W, Savani BN, Veys PA, Bredeson CN, Giralt SA, LeMaistre CF (2015) Indications for autologous and allogeneic hematopoietic cell transplantation: guidelines from the American Society for Blood and Marrow Transplantation. Biol Blood Marrow Transplant 21(11):1863–1869. 10.1016/j.bbmt.2015.07.032 26256941 10.1016/j.bbmt.2015.07.032 PMC4830270

[10] Villafuerte KRV, Martinez CJH, Dantas FT, Carrara HHA, Dos Reis FJC, Palioto DB (2018) The impact of chemotherapeutic treatment on the oral microbiota of patients with cancer: a systematic review. Oral Surg Oral Med Oral Pathol Oral Radiol 125(6):552–566. 10.1016/j.oooo.2018.02.008 29566996 10.1016/j.oooo.2018.02.008

[11] Chiusolo P, Metafuni E, Sterbini FP, Giammarco S, Masucci L, Leone G, Sica S (2015) Gut microbiome changes after stem cell transplantation. Blood 126:1953. 10.1182/blood.V126.23.1953.1953

[12] Scordo M, Shah GL, Adintori PA, Knezevic A, Devlin SM, Buchan ML, Preston EV, Lin AP, Rodriguez NT, Carino CA, Nguyen LK, Sitner NC, Barasch A, Klang MG, Maloy MA, Mastrogiacomo B, Carlow DC, Schofield RC, Slingerland AE, Slingerland JB, Stein-Thoeringer CK, Lahoud OB, Landau HJ, Chung DJ, van den Brink MRM, Peled JU, Giralt SA (2022) A prospective study of dysgeusia and related symptoms in patients with multiple myeloma after hematopoietic cell transplantation. Cancer 128(21):3850–3859. 10.1002/cncr.34444 36041227 10.1002/cncr.34444 PMC10010839

[13] Okada N, Hanafusa T, Aba S, Sato C, Nakamura T, Teraoka K, Abe M, Kawazoe K, Ishizawa K (2016) Evaluation of the risk factors associated with high dose chemotherapy-induced dysgeusia in patients undergoing autologous hematopoietic stem cell transplantation: possible usefulness of cryotherapy in dysgeusia prevention. Support Care Cancer 24(9):3979–3985. 10.1007/s00520-016-3244-9 27129837 10.1007/s00520-016-3244-9

[14] Scordo M, Shah G, Preston E, Rodriguez N, Lin A, Maloy M, Mastrogiacomo B, Carlow D, Schofield R, Knezevic A, Devlin SM, Landau HJ, Chung DJ, Peled JU, Buchan ML, Giralt S (2018) Dysgeusia is associated with higher melphalan pharmacokinetic levels and results in poorer caloric intake and worse symptom burden after autologous stem cell transplantation for multiple myeloma. Blood 132:2136. 10.1182/blood-2018-99-114715

[15] Slavik M, Wu J, Riley C (1993) Salivary excretion of anticancer drugs. Ann N Y Acad Sci 694:319–321. 10.1111/j.1749-6632.1993.tb18377.x 8215078 10.1111/j.1749-6632.1993.tb18377.x

[16] Pereira MR, Pouch SM, Scully B (2018) Infections in allogeneic stem cell transplantation. In: Safdar A (ed) Principles and practice of transplantation infectious diseases. Springer, New York, NY, pp 209–226. 10.1007/978-1-4939-9034-4_11

[17] Hull KM, Kerridge I, Schifter M (2012) Long-term complications of allogeneic SCT. Bone Marrow Transplant 47(2):265–270. 10.1038/bmt.2011.63 21441960 10.1038/bmt.2011.63

[18] Scordo M, Shah G, Peled J, Preston E, Buchan M, Epstein J, Barasch A, Giralt S (2018) Unlocking the complex flavors of dysgeusia after hematopoietic cell transplantation. Biol Blood Marrow Transplant 24(3):425–432. 10.1016/j.bbmt.2017.10.022 29051023 10.1016/j.bbmt.2017.10.022 PMC6712422

[19] Brennan MT, Hasséus B, Hovan AJ, Raber-Durlacher JE, Blijlevens NM, Huysmans MC, Garming Legert K, Johansson JE, Moore CG, von Bültzingslöwen I (2018) Impact of oral side effects from conditioning therapy before hematopoietic stem cell transplantation: protocol for a multicenter study. JMIR Res Protoc 7(4):e103. 10.2196/resprot.8982 29685874 10.2196/resprot.8982 PMC5938569

[20] Kaur K, Sculley D, Veysey M, Lucock M, Wallace J, Beckett EL (2021) Bitter and sweet taste perception: relationships to self-reported oral health habits and oral health status in a survey of Australian adults. BMC Oral Health 21(1):553. 10.1186/312903-021-01920 34715836 10.1186/s12903-021-01910-8 PMC8555166

[21] Schertel Cassiano L, Ribeiro AP, Peres MA, Lopez R, Fjaeldstad A, Marcini l, Nascimento GG (2024) Self-reported periodontitis association with impaired smell and taste: a multicenter survey. Oral Dis 30(3):1516–1524. 10.1111/odi.4601 37114436 10.1111/odi.14601

[22] World Health Organization (1979) WHO handbook for reporting results of cancer treatment. https://iris.who.int/handle/10665/37200. Accessed 14 April 2025

[23] Caplan DJ, Hunt RJ (1996) Salivary flow and risk of tooth loss in an elderly population. Community Dent Oral Epidemiol 24(1):68–71. 10.1111/j.1600-0528 8833518 10.1111/j.1600-0528.1996.tb00816.x

[24] Skallsjö K, von Bültzingslöwen I, Hasséus B, Johansson JE, Öhman J, Raber-Durlacher JE, Huysmans MDNJM, Laheij AMGA, van Leeuwen SJM, Hovan AJ, Garming Legert K, Nguyen HM, Turk PJ, Rozema FR, Blijlevens NMA, Brennan MT (2023) Oral health in patients scheduled for hematopoietic stem cell transplantation in the Orastem study. PLoS One 18(5):e0285615. 10.1371/journal.pone.0285615 37200298 10.1371/journal.pone.0285615 PMC10194987

[25] Jensen SB, Vissink A (2014) Salivary gland dysfunction and xerostomia in Sjögren’s syndrome. Oral Maxillofac Surg Clin North Am 26(1):35–53 24287192 10.1016/j.coms.2013.09.003

[26] Mays JW, Fassil H, Edwards DA, Pavletic SZ, Bassim C (2013) Oral chronic graft-versus-host disease: current pathogenesis, therapy and research. Oral Dis 19(4):327–346. 10.1111/odi.12028 23107104 10.1111/odi.12028 PMC3561479

[27] Boor M, Raber-Durlacher JE, Hazenberg MD, Rozema FR, Laheij AMGA (2022) Taste and smell disturbances in patients with chronic oral graft vs host disease: an observational study. Front Oral Health 3:934607. 10.3389/froh.2022.934607 36160117 10.3389/froh.2022.934607 PMC9500145

[28] Dean D, Sroussi H (2022) Chronic oral graft-versus-host disease. Front Oral Health 3:903154. 10.3389/froh.2022.903154 35719318 10.3389/froh.2022.903154 PMC9205403

[29] Boer CC, Correa ME, Miranda EC, de Souza CA (2010) Taste disorders and oral evaluation in patients undergoing allogeneic hematopoietic SCT. Bone Marrow Transplant 45(4):705–711. 10.1038/bmt.2009.237 19767788 10.1038/bmt.2009.237

[30] Abasaeed R, Coldwell SE, Lloid ME, Soliman SH, Macris PC, Schubert MM (2018) Chemosensory changes and quality of life in patients undergoing hematopoietic stem cell transplantation. Support Care Cancer 26(10):3553–3561. 10.1007/s00520-018-4200-7 29704111 10.1007/s00520-018-4200-7 PMC6117208

[31] Marinone MG, Rizzone D, Ferremi P, Rossi G, Izzi T, Brusotti C (1991) Late taste disorders in bone marrow transplantation: clinical evaluation with taste solutions in autologous and allogeneic bone marrow recipients. Haematologica 76(6):519–522 1820992

[32] Do Nascimento ML, Farias AB, Carvalho AT, Albuquerque RF, Ribeiro LN, Leao JC, Silva IH (2019) Impact of xerostomia on the quality of life of patients submitted to head and neck radiotherapy. Med Oral Patol Oral Cir Bucal 24(6):e770–e775. 10.4317/medoral.23131 31655838 10.4317/medoral.23131 PMC6901149

[33] Logemann JA, Pauloski BR, Rademaker AW, Lazarus CL, Mittal B, Gaziano J, Stachowiak L, MacCracken E, Newman LA (2023) Xerostomia: 12-month changes in saliva production and its relationship to perception and performance of swallow function, oral intake, and diet after chemoradiation. Head Neck 25(6):432–437. 10.1002/hed.10255 10.1002/hed.10255 12784234

[34] Bezzina O, Gallagher P, Mitchell S, Bowman SJ, Griffiths B, Hindmarsh V, Hargreaves B, Price EJ, Pease CT, Emery P, Lanyon P, Bombardieri M, Sutcliffe N, Pitzalis C, Hunter J, Gupta M, McLaren J, Cooper AM, Regan M, Giles IP, Isenberg DA, Saravanan V, Coady D, Dasgupta B, McHugh NJ, Young-Min SA, Moots RJ, Gendi N, Akil M, MacKay K, Ng WF, Robinson LJ, UK Primary Sjögren’s Syndrome Registry (2017) Subjective and objective measures of dryness symptoms in primary Sjögren’s syndrome: capturing the discrepancy. Arthritis Care Res (Hoboken) 69(11):1714–1723. 10.1002/acr.23165 27992710 10.1002/acr.23165 PMC5698764

[35] Fox PC, Busch KA, Baum BJ (1987) Subjective reports of xerostomia and objective measures of salivary gland performance. J Am Dent Assoc 115(4):581–584 3477595 10.1016/s0002-8177(87)54012-0

[36] Thorne T, Olson K, Wismer W (2015) A state-of-the-art review of the management and treatment of taste and smell alterations in adult oncology patients. Support Care Cancer 23(9):2843–2851. 10.1007/s00520-015-2827-1 26162535 10.1007/s00520-015-2827-1

