# Supplementary material for: Longitudinal assessment and predictors of subjective taste change after hematopoietic cell transplantation (HCT)
Source: Support Care Cancer. 2026 Jun 3;34(6):605. doi: 10.1007/s00520-026-10831-7 (PMC13233654; doi:10.1007/s00520-026-10831-7)
Supplement: Supplementary file 2 — (DOCX 14.4 KB) [file 520_2026_10831_MOESM2_ESM.docx]

**Supplement 2: Follow-Up Visits (allogeneic patients only). Severity of taste change during the past 24 hours on a 4-point scale (“not at all”, “ a little”, “quite a bit” or “very much”**

|  | **3 months** | | **6 months** | | **12 months** | |
| --- | --- | --- | --- | --- | --- | --- |
| **Variable** | **Not at all/a little** | **Quite a bit/ very much** | **Not at all/a little** | **Quite a bit/ very much** | **Not at all/a little** | **Quite a bit/ very much** |
|  |  |  |  |  |  |  |
| **N** | 91 | 28 | 90 | 18 | 84 | 12 |
| Chronic oral GVHD |  |  |  |  |  |  |
| No N (%) | 82 (78.1) | 23 (21.9) | 69 (87.3) | 10 (12.7) | 65 (89.0) | 8 (11.0) |
| Yes N (%) | 9 (64.3) | 5 (35.7) | 21 (72.4) | 8 (27.6) | 19 (82.6) | 4 (17.4) |
|  | p=0.31 | | p=0.08 | | p=0.47 | |
