# Supplementary material for: Longitudinal assessment and predictors of subjective taste change after hematopoietic cell transplantation (HCT)
Source: Support Care Cancer. 2026 Jun 3;34(6):605. doi: 10.1007/s00520-026-10831-7 (PMC13233654; doi:10.1007/s00520-026-10831-7)
Supplement: Supplementary file 3 — (DOCX 18.3 KB) [file 520_2026_10831_MOESM3_ESM.docx]

| **Supplement 3: Factors Predictive of Severe Taste Change after Hematopoietic Cell Transplantation (HCT) at Different Follow-Up Periods*** | | | | | | |
| --- | --- | --- | --- | --- | --- | --- |
|  | **3-months**  **(autos and allos)** | | **6-months**  **(allos only)** | | **12-months**  **(autos and allos)** | |
|  | **Odds Ratio (95% CI)** | **p- value** | **Odds Ratio (95% CI)** | **p-value** | **Odds Ratio (95% CI)** | **p-value** |
| Age at baseline: 5-year increase | 1.3 (1.1, 1.5) | **0.0015** | 1.3 (1.1, 1.7) | **0.0070** | 1.6 (1.1, 2.4) | **0.0145** |
| Gender: female vs male | 1.3 (0.6, 2.7) | 0.4687 | 1.2 (0.4, 3.6) | 0.7479 | 2.7 (0.8, 9.1) | 0.0972 |
| Type of transplant: allogeneic vs autologous | 2.2 (1.1, 4.6) | **0.0365** | N/A | N/A | 1.2 (0.2, 5.5) | 0.8593 |
| Regimen: myeloablative vs reduced intensity/non-myeloablative conditioning | 1.7 (0.5, 5.8) | 0.4128 | 2.1 (0.5, 8.9) | 0.3156 | 0.6 (0.1, 2.6) | 0.5083 |
| WHO mucositis: < Grade 2 vs ≥ Grade 2 | 1.1 (0.5, 2.4) | 0.7550 | 0.8 (0.2, 2.8) | 0.7373 | 1.0 (0.3, 3.4) | 0.9863 |
| Plaque score: < 20% vs > 20% | 1.8 (0.7, 4.6) | 0.2602 | 0.9 (0.3, 2.8) | 0.8659 | 1.9 (0.4, 7.7) | 0.3970 |
| Salivary flow pre-HCT: <1 mL/min vs >1 mL/ min | 1.3 (0.6, 2.7) | 0.5612 | 0.7 (0.2, 2.1) | 0.4925 | 2.2 (0.7, 6.8) | 0.1861 |
| Brushing habits: < 2x/day vs > 2x/day | 1.2 (0.5, 2.7) | 0.7450 | 0.4 (0.1, 1.1) | 0.0694 | 1.9 (0.4, 9.4) | 0.4211 |
| Flossing habits: < once per day vs > once per day | 1.7 (0.8, 3.8) | 0.1744 | 0.6 (0.2, 1.8) | 0.3315 | 0.9 (0.3, 2.9) | 0.8214 |
| Dental visits: never/acute problems vs routinely | 0.8 (0.3, 1.8) | 0.5298 | 1.9 (0.6, 6.0) | 0.2767 | 1.4 (0.4, 4.9) | 0.5645 |
| Prior chemotherapy: Yes vs No | 1.0 (0.2, 5.3) | 0.9762 | 1.1 (0.1, 8.1) | 0.9447 | 0.7 (0.1, 6.5) | 0.7460 |
| Taste change from prior chemotherapy: Yes vs No | 1.7 (0.8, 3.4) | 0.1677 | 1.1 (0.4, 3.1) | 0.9257 | 1.4 (0.4, 4.9) | 0.5560 |
| Dry mouth from prior chemotherapy: Yes vs No | 1.5 (0.7, 3.1) | 0.3343 | 1.2 (0.4, 3.6) | 0.7823 | 1.9 (0.6, 6.2) | 0.3021 |
| Mucositis from prior chemotherapy: Yes vs No | 1.4 (0.5, 4.2) | 0.5642 | 1.8 (0.5, 6.4) | 0.3737 | 1.2 (0.3, 5.0) | 0.7805 |
| Taste change during hospitalization: Yes vs No | 1.3 (0.6, 3.0) | 0.4786 | 1.1 (0.3, 3.9) | 0.8574 | 1.7 (0.5, 6.1) | 0.4279 |

* Autologous patients were followed up in person and by questionnaire at 3 months; by questionnaire only at 12 months. Allogeneic patients were followed up in person and by questionnaire at all 3 follow-up periods.
